# X-linked Hypophosphatemic Rickets Revealed by Exome Sequencing: A Pediatric Case Report of a PHEX Pathogenic Variant

**DOI:** 10.7759/cureus.109583

**Published:** 2026-05-25

**Authors:** Karima Larbi Ouassou, Hassani Amale, Rachid Abilkassem

**Affiliations:** 1 Pediatric Medicine, Mohammed V Military Training Hospital, Rabat, MAR

**Keywords:** exome sequencing, fgf23, hypophosphatemia, pediatrics, phex gene, varus deformity, xlh, x-linked hypophosphatemic rickets

## Abstract

X-linked hypophosphatemic rickets (XLH) is the most common form of hereditary vitamin-resistant rickets, caused by inactivating mutations of the PHEX (phosphate-regulating endopeptidase homolog, X-linked) gene, leading to elevated fibroblast growth factor 23 (FGF23) and chronic hypophosphatemia secondary to renal phosphate wasting.

We report the case of a 3.5-year-old boy presenting with bilateral varus deformity of the lower limbs, frontal bossing, rachitic rosary, and growth retardation (-2.5 SD). Laboratory investigations revealed hypophosphatemia (0.88 mmol/L) with severely reduced urinary phosphate excretion (196.8 mg/24 h), elevated alkaline phosphatase (521 IU/L), and normal serum calcium and vitamin D levels. Exome sequencing identified a hemizygous pathogenic variant in PHEX: c.2192T>G (p.Phe731Cys), confirming the diagnosis of XLH.

This case illustrates the decisive contribution of exome sequencing in confirming vitamin-resistant rickets and the importance of early diagnosis to prevent irreversible orthopedic sequelae.

## Introduction

Rickets is a disorder of impaired bone mineralization occurring in growing children, manifesting as skeletal deformities, growth retardation, and bone pain. Among hereditary forms, X-linked hypophosphatemic rickets (XLH; OMIM: 307800) is the most prevalent, with an estimated incidence of 1/20,000 live births, following an X-linked dominant inheritance pattern [1]. XLH results from inactivating mutations in the PHEX gene (phosphate-regulating endopeptidase homolog, X-linked), located at Xp22.11, leading to fibroblast growth factor 23 (FGF23) accumulation, renal phosphate wasting, and impaired calcitriol synthesis [2,3].

The differential diagnosis of rickets in pediatric practice includes several etiologies with overlapping clinical presentations. Nutritional rickets, caused by vitamin D deficiency, remains the most common form globally and typically responds to vitamin D supplementation. Hereditary forms include vitamin D-dependent rickets (VDDR) types 1 and 2, caused by mutations in CYP27B1 (1α-hydroxylase deficiency) and VDR (vitamin D receptor defects), respectively; hereditary hypophosphatemic rickets with hypercalciuria (HHRH) due to SLC34A3 mutations; and autosomal dominant or recessive hypophosphatemic rickets related to FGF23 pathway dysregulation (e.g., FGF23, DMP1, ENPP1 mutations). Tumor-induced osteomalacia (TIO), though rare in children, must be considered when FGF23 levels are elevated. XLH is distinguished from these entities by normal or elevated vitamin D levels (unlike nutritional rickets and VDDR), renal phosphate wasting with appropriately low urinary calcium (unlike HHRH), resistance to standard vitamin D therapy, and X-linked dominant inheritance [4,5].

We report the case of a 3.5-year-old Moroccan boy in whom the diagnosis of XLH was confirmed by whole-exome sequencing, revealing a hemizygous pathogenic variant, PHEX c.2192T>G (p.Phe731Cys). This case illustrates the added value of next-generation genomic technologies when integrated with systematic clinical investigation in the diagnostic workup of hereditary rickets in resource-limited settings.

## Case presentation

Patient history

The patient is a three-year-and-six-month-old male child (born August 27, 2022), born to non-consanguineous parents with no family history of rickets or known hereditary bone disease. His personal medical history was unremarkable.

Developmental Milestones

Motor development was within normal ranges: head control at four months, sitting without support at seven months, standing with support at 12 months, and independent walking at 15 months. Language development progressed appropriately, with first words at 12 months and age-appropriate vocabulary expansion. Cognitive and social development showed normal interaction, with no developmental delay noted.

Clinical Course

Skeletal deformities first became noticeable at approximately 15 months of age, coinciding precisely with the onset of independent ambulation. Parents initially observed progressive bilateral bowing of the lower limbs that worsened over subsequent months. The patient received oral vitamin D supplementation (standard dose for nutritional rickets) for several months without clinical improvement, prompting further specialized investigation. Growth retardation became progressively apparent during the second year of life, with the child falling below the third percentile (-2.5 SD) by three years of age. During this same period, frontal bossing and rachitic rosary were observed by parents, along with the development of a characteristic waddling gait as ambulation progressed. Specialized pediatric evaluation was sought at three years and six months of age due to persistence and worsening of skeletal deformities despite standard vitamin D therapy.

Clinical examination

The child was in good general condition. Anthropometric assessment showed a weight of 13 kg and a height of 86 cm (-2.5 SD), indicating significant growth retardation. Musculoskeletal examination revealed bilateral asymmetric lower limb varus deformities, frontal bossing, palpable rachitic rosary on thoracic examination, and a waddling gait. No craniotabes or wrist epiphyseal enlargement were detected.

Hearing Assessment

Otoscopic examination revealed normal tympanic membranes bilaterally, with no middle ear effusion. Age-appropriate behavioral audiometry was performed, and no hearing loss was detected. Sensorineural hearing loss is not a typical feature of XLH but has been occasionally reported in severe cases or with long-term bisphosphonate use; our patient showed no auditory impairment.

Laboratory investigations

Laboratory results are summarized in Table 1. The biochemical profile was consistent with XLH, featuring profound hypophosphatemia with massive renal phosphate wasting, markedly elevated alkaline phosphatase, normal serum calcium and vitamin D levels, and high-normal parathyroid hormone (PTH).

**Table 1:** Summary of laboratory investigations. Symbols: ↓ = below normal range; ↑ = above normal range; ↓↓ = markedly below normal range; ↑↑ = markedly above normal range. Abbreviations: PTH, Parathyroid Hormone; TRP, Tubular Reabsorption of Phosphate; TmP/GFR, Tubular Maximum Reabsorption of Phosphate per Glomerular Filtration Rate; UCa, Urinary Calcium; FGF23, Fibroblast Growth Factor 23; ALT, Alanine Aminotransferase; AST, Aspartate Aminotransferase; GGT, Gamma-Glutamyltransferase.

Parameter	Result	Reference Range
Serum phosphate	0.88 mmol/L ↓	0.89-1.28 mmol/L
Alkaline phosphatase	521 IU/L ↑	<350 IU/L
Serum calcium	2.31 mmol/L	2.2-2.62 mmol/L
25-OH vitamin D	48.9 ng/mL	30-80 ng/mL
Intact PTH	61.88 pg/mL	15-65 pg/mL
Urinary phosphate (24h)	196.8 mg/24h ↓↓	650-1400 mg/24h
Urinary calcium (24h)	8 mg/24h	<300 mg/24h
TRP (tubular reabsorption of phosphate)	78% ↓	>85%
TmP/GFR	0.65 mmol/L ↓	>1.15 mmol/L
UCa/Creatinine ratio	0.05 mg/mg	<0.2 mg/mg
FGF23 (C-terminal)	289 RU/mL ↑↑	8-54 RU/mL
Serum creatinine	0.35 mg/dL	Normal for age
Urinalysis (glucose)	Negative	Negative
Urinalysis (protein)	Negative	Negative
Serum bicarbonate	22 mEq/L	22-28 mEq/L
ALT	28 IU/L	<40 IU/L
AST	32 IU/L	<40 IU/L
GGT	8 IU/L	3-22 IU/L
Hemoglobin	12.4 g/dL	12.5-14.5 g/dL

Phosphate Handling Parameters

Tubular reabsorption of phosphate (TRP) was calculated at 78% (normal >85% for age), and tubular maximum reabsorption of phosphate normalized to glomerular filtration rate (TmP/GFR) was 0.65 mmol/L (normal >1.15 mmol/L for age), quantitatively confirming severe renal phosphate wasting characteristic of XLH [2].

Hepatic and Renal Function

Baseline liver function tests were normal (ALT: 28 IU/L, AST: 32 IU/L, GGT: 8 IU/L), confirming no hepatic pathology. Serum creatinine was 0.35 mg/dL (normal for age), with calculated eGFR >90 mL/min/1.73 m², confirming no chronic kidney disease.

Exclusion of Fanconi Syndrome

Urinalysis showed no glucosuria (glucose: negative), no proteinuria (protein: negative; urinary protein/creatinine ratio: 0.08 mg/mg, normal), and no metabolic acidosis (serum bicarbonate: 22 mEq/L, normal). These findings exclude generalized proximal tubulopathy (Fanconi syndrome) and confirm isolated renal phosphate wasting characteristic of XLH.

Urinary Calcium/Creatinine Ratio

Urinary calcium/creatinine ratio = 0.05 mg/mg (reference <0.2), confirming appropriately low urinary calcium excretion, consistent with secondary hyperparathyroidism and distinguishing XLH from HHRH.

Serum FGF23

FGF23 (C-terminal) = 289 RU/mL (reference: 8-54 RU/mL), confirming markedly elevated FGF23, the pathophysiological hallmark of XLH caused by PHEX deficiency [3].

Hypocalcemia

Serum calcium was normal (2.31 mmol/L) throughout the clinical course, with no clinical signs of hypocalcemia observed (no tetany, carpopedal spasm, Chvostek or Trousseau signs, seizures, or prolonged QTc on ECG). This finding is consistent with XLH, where hypocalcemia is typically not a feature (unlike VDDR or hypoparathyroidism), as PTH remains functional and serum calcium is actively maintained.

Radiological assessment

**Figure 1:**
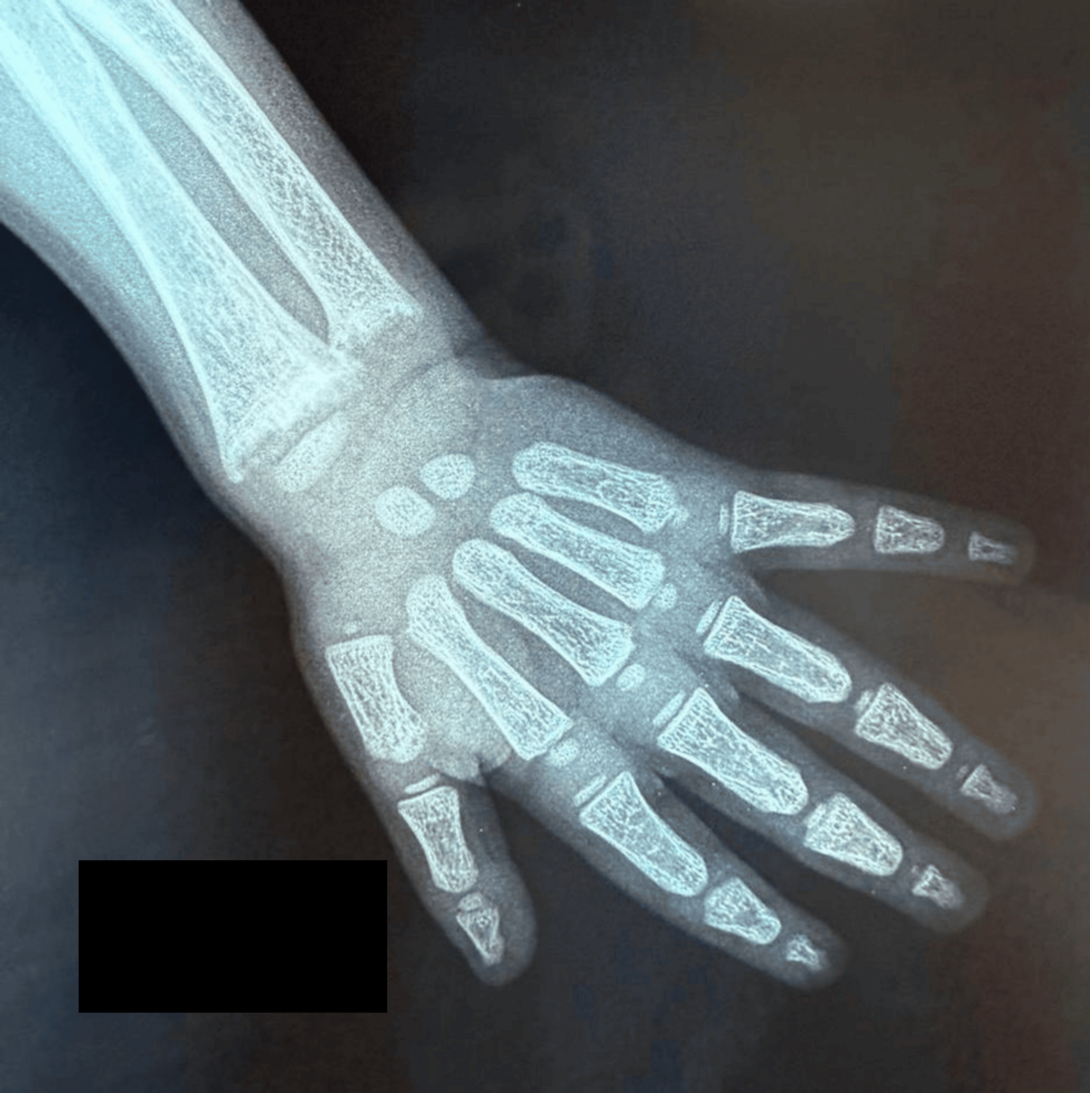
Hand and left wrist radiograph for bone age assessment. Ossification centers are compatible with chronological age. No significant bone maturation delay. Metaphyseal changes consistent with active rickets are noted.

**Figure 2:**
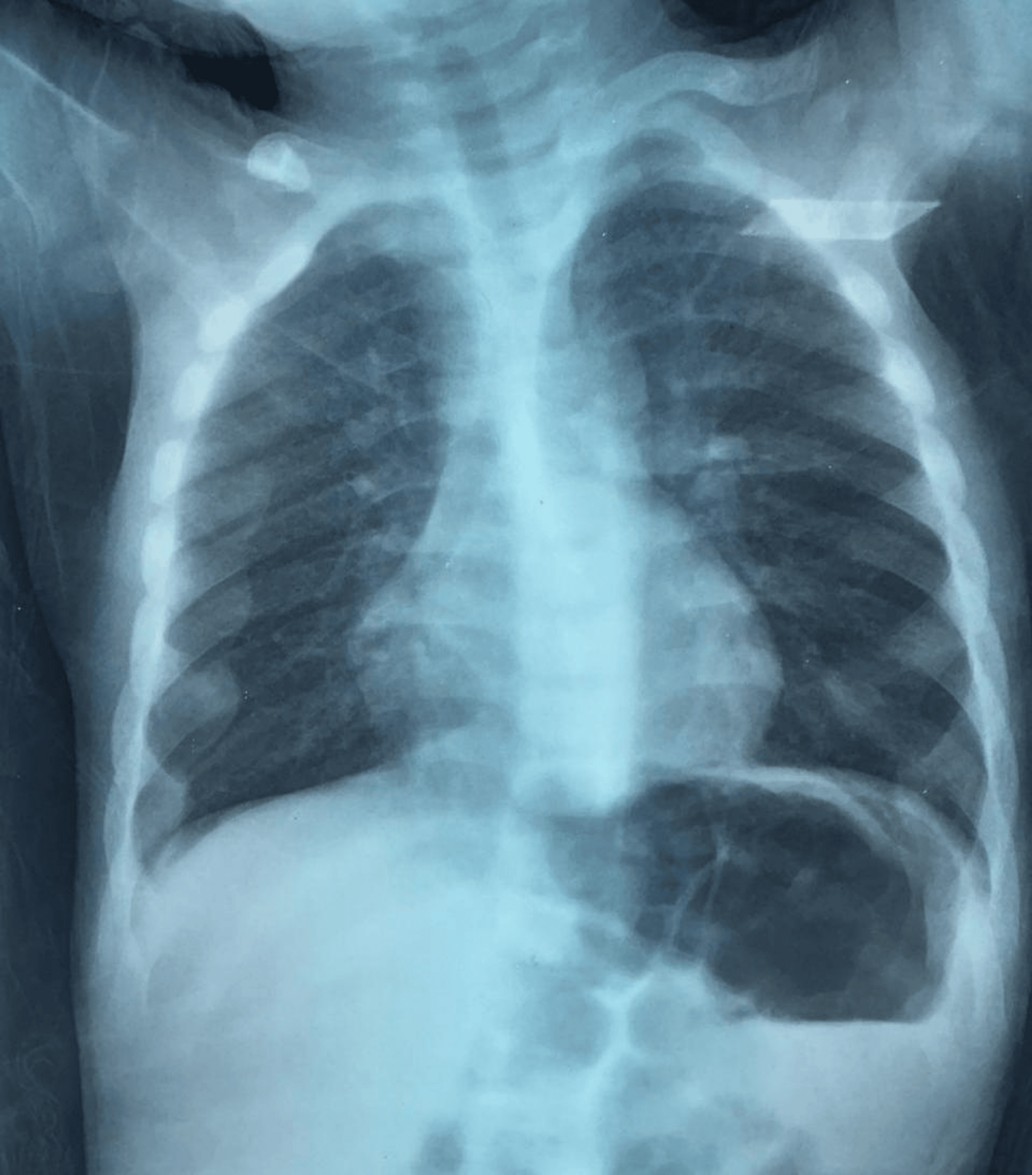
Anteroposterior chest radiograph showing deformity of the right middle costal arches consistent with rachitic rosary appearance, indicating active rickets. Cardiomediastinal silhouette is normal. No pleural effusion.

**Figure 3:**
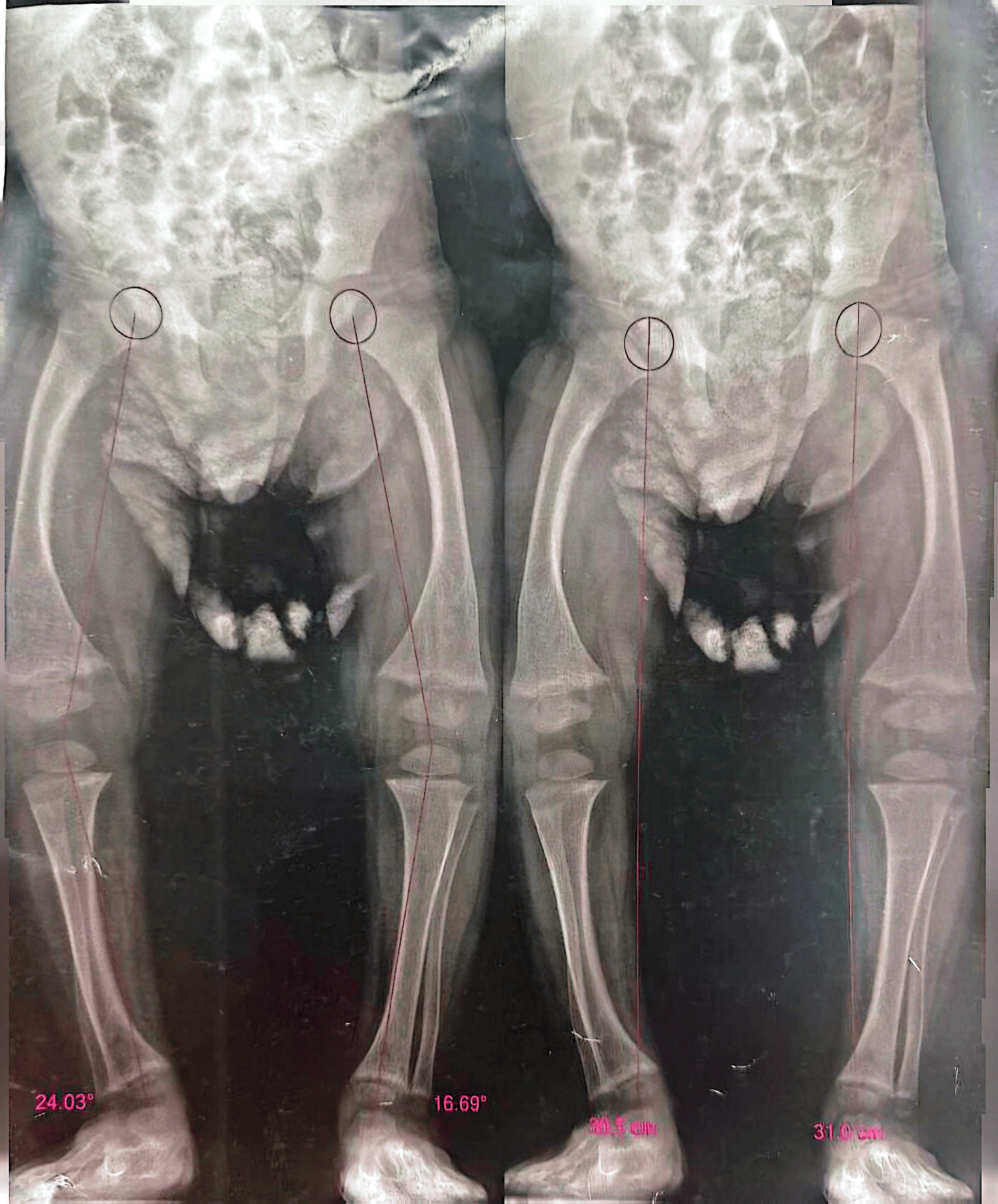
Lower limb goniometry demonstrating bilateral varus deformities. Mechanical axis deviation angle (DAC): right = 24.03° (26.3° of varus), left = 16.69° (18.69° of varus). Total limb lengths: right = 30.5 cm, left = 31.0 cm. Bilateral coxa valga is noted.

Hand and wrist radiograph showed ossification centers compatible with chronological age, with no significant bone maturation delay (Figure 1). Metaphyseal changes consistent with active rickets were noted. Chest radiograph revealed probable deformity of the right middle costal arches producing a rachitic rosary pattern, with normal cardiomediastinal silhouette (Figure 2). Lower limb goniometry confirmed bilateral varus deformities, with a mechanical axis deviation angle (DAC) of 24.03° on the right (26.3° of varus) and 16.69° on the left (18.69° of varus). Lower limb lengths were 30.5 cm (right) and 31.0 cm (left) (Figure 3). Bilateral coxa valga was also noted on the pelvic radiograph [4].

Exclusion of Chondrodysplasias

Radiographic findings were inconsistent with skeletal dysplasias: normal skeletal proportions with no rhizomelic, mesomelic, or acromelic shortening; normal epiphyseal and metaphyseal architecture on long bone radiographs (excluding rachitic changes); no vertebral anomalies, such as platyspondyly, coronal clefting, or anterior beaking; normal skull base and craniovertebral junction; absence of dysplastic features, such as flared metaphyses (beyond rickets), cone-shaped epiphyses, or stippled epiphyses; and bone age appropriate for chronological age (unlike many skeletal dysplasias with delayed or advanced bone maturation). Clinical findings also excluded skeletal dysplasia syndromes: normal neurodevelopment and cognition, no dysmorphic facial features, no joint laxity or contractures, and no organomegaly. These findings, combined with the biochemical phenotype of isolated renal phosphate wasting, effectively ruled out primary chondrodysplasias (achondroplasia, hypochondroplasia, pseudoachondroplasia, etc.).

Genetic analysis

Rationale for Genetic Testing

Whole-exome sequencing was pursued based on several clinical considerations: failure of conventional vitamin D therapy despite adequate supplementation; biochemical profile highly suggestive of hereditary hypophosphatemic rickets (isolated renal phosphate wasting with normal vitamin D and calcium levels); absence of nutritional deficiency or malabsorption, ruling out nutritional rickets; suspected X-linked dominant inheritance pattern based on phenotype (male patient, no family history, suggesting possible maternal mosaicism or de novo mutation); and the need for a definitive molecular diagnosis to guide targeted therapy (potential burosumab eligibility) and provide accurate genetic counseling.

Whole-exome sequencing (Hub for Precision Medicine, HMIMV-Rabat; report dated 05/03/2026) returned a POSITIVE result. A hemizygous pathogenic variant was identified in the PHEX gene: c.2192T>G (p.Phe731Cys). Full characteristics are presented in Table 2.

**Table 2:** Characteristics of the pathogenic variant identified in the PHEX gene. Abbreviations: PHEX, Phosphate-Regulating Endopeptidase Homolog X-Linked; XLH, X-Linked Hypophosphatemic Rickets; OMIM, Online Mendelian Inheritance in Man; REVEL, Rare Exome Variant Ensemble Learner.

Parameter	Result
Gene	PHEX (Phosphate-regulating Endopeptidase Homolog, X-linked)
Genomic position	X-22247895-T-G (GRCh38)
cDNA variant	NM_000444.6:c.2192T>G
Protein variant	NP_000435.3:p.Phe731Cys
Mutation type	Missense
Zygosity	Hemizygous
Classification	Pathogenic
Associated disease	XLH - X-linked dominant hypophosphatemic rickets (OMIM: 307800)
gnomAD v4.1.0	Absent (not reported in the general population)
REVEL score	0.96 (≥0.6 = pathogenic)
3Cnet score	0.90 (>0.75 = pathogenic)
ClinVar ID	VCV000951425 (pathogenic)
Inheritance	X-linked dominant - maternal status under evaluation
Report date	05/03/2026 - Hub for Precision Medicine, HMIMV-Rabat

This missense substitution (p.Phe731Cys) affects a critical functional domain of the PHEX metalloendopeptidase, impairing enzymatic activity and driving FGF23 accumulation. The variant is absent from gnomAD v4.1.0, is unanimously predicted deleterious by in silico tools (REVEL: 0.96; 3Cnet: 0.90), and has been previously classified as pathogenic in ClinVar (VCV000951425) [5,6].

Exclusion of secondary hypophosphatemic disorders

A systematic investigation was performed to exclude secondary causes of FGF23-mediated hypophosphatemia:

*Tumor-Induced Osteomalacia* *(TIO)*

Clinical examination revealed no palpable masses or cutaneous lesions. Systematic imaging was not performed given the patient's young age (3.5 years) and the extremely low prevalence of TIO in early childhood (typically presents after age 20 years). Clinical follow-up is planned.

Cutaneous Skeletal Hypophosphatemia Syndrome (CSHS)

Comprehensive dermatological examination showed no epidermal nevi, sebaceous nevi, pigmented lesions, café-au-lait macules, or other neurocutaneous stigmata. CSHS was explicitly excluded based on the absence of characteristic skin findings.

McCune-Albright Syndrome

Skeletal imaging showed no fibrous dysplasia. No café-au-lait spots with irregular "coast of Maine" borders were present. No precocious puberty or other endocrinopathies were observed.

Neurofibromatosis Type 1

No neurofibromas, axillary or inguinal freckling, or Lisch nodules were identified. Family history was negative.

Treatment and management

Conventional therapy was initiated: oral phosphate supplementation (three daily doses) and alfacalcidol (1-alpha-hydroxyvitamin D), with quarterly biochemical monitoring and biannual radiological follow-up [7]. Genetic counseling was arranged, with maternal genetic testing recommended to determine whether the variant was de novo or inherited.

Treatment Response and Follow-Up

At the six-month follow-up, the patient demonstrated clinical and biochemical improvement: (i) Biochemical response: Serum phosphate improved to 1.02 mmol/L (from 0.88 mmol/L at baseline). Alkaline phosphatase decreased to 412 IU/L (from 521 IU/L), indicating improved bone mineralization. (ii) Radiological improvement: Follow-up wrist radiograph showed partial metaphyseal healing with decreased cupping and fraying of growth plates. (iii) Growth response: Growth velocity improved, with the patient gaining 3.2 cm over six months (annualized rate: 6.4 cm/year), representing catch-up growth compared to pre-treatment velocity. (iv) Ongoing management: The patient continues on conventional therapy with regular monitoring. Orthopedic surgical planning for correction of varus deformities is under consideration, pending further skeletal maturation and response to medical therapy.

## Discussion

**Figure 4:**
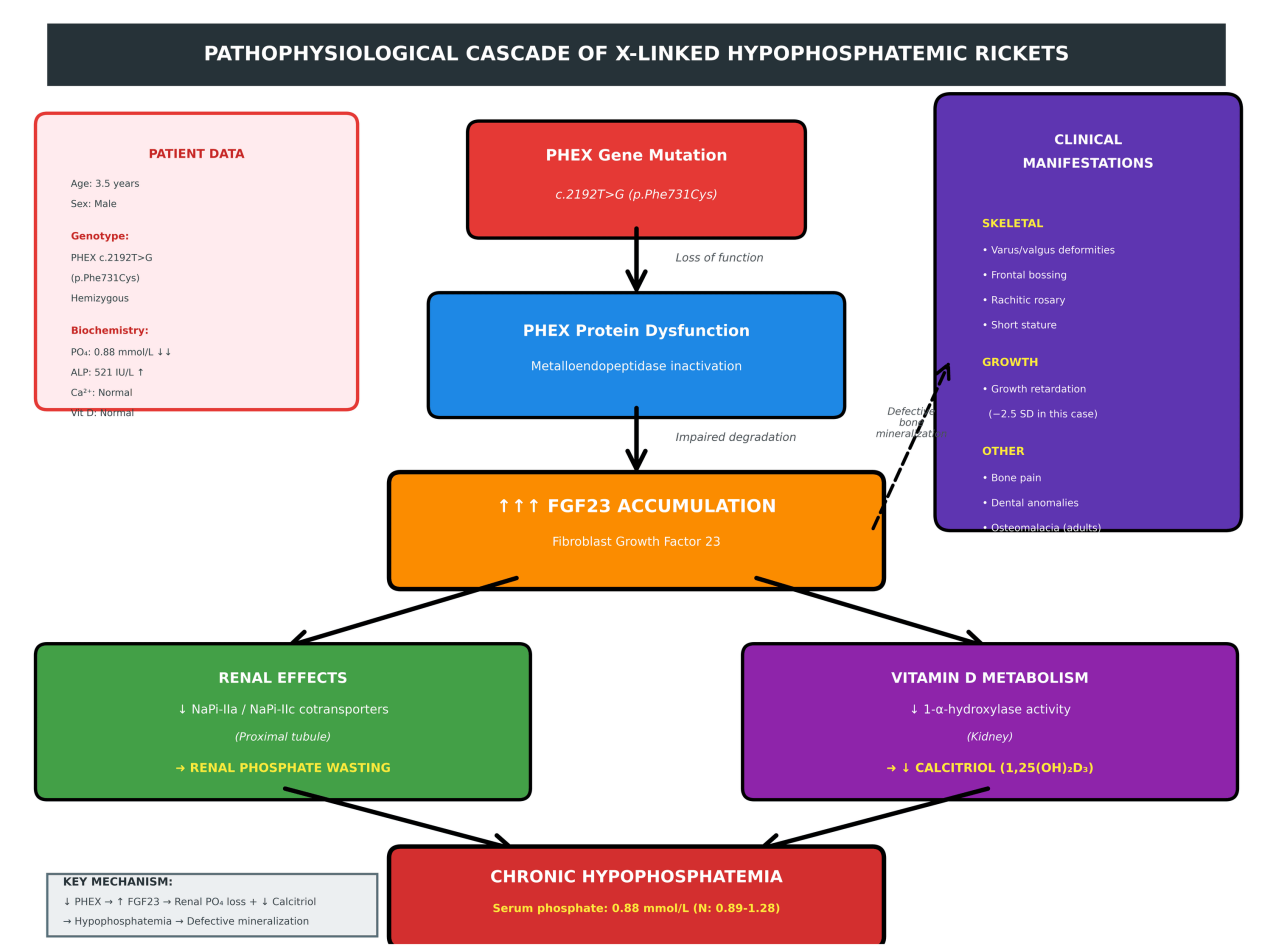
Pathophysiological cascade in the current patient with X-linked hypophosphatemic rickets (XLH). The identified PHEX (phosphate-regulating endopeptidase homolog, X-linked) variant (c.2192T>G; p.Phe731Cys) leads to fibroblast growth factor 23 (FGF23) accumulation, causing renal phosphate wasting and reduced calcitriol synthesis, resulting in chronic hypophosphatemia and defective bone mineralization with clinical manifestations of rickets.

XLH results from inactivating mutations of PHEX, driving FGF23 accumulation, which in turn inhibits renal NaPi-IIa/IIc cotransporters and 1-alpha-hydroxylase, causing phosphate wasting and calcitriol deficiency [3,8]. The resulting chronic hypophosphatemia impairs osteoid mineralization, giving rise to the phenotype observed in our patient (Figure 4).

The clinical presentation is characteristic of pediatric XLH, comprising the four cardinal features: varus deformities appearing at ambulation onset (15 months), growth retardation (-2.5 SD), frontal bossing, and rachitic rosary. Imaging documented the severity of deformities, with marked bilateral varus (26.3° right and 18.69° left) and coxa valga deformities, exposing the patient to permanent orthopedic sequelae in the absence of early, effective treatment [4,9].

The identified variant, PHEX c.2192T>G (p.Phe731Cys), is a hemizygous missense mutation absent from population databases, unanimously predicted to be deleterious, and previously reported in ClinVar. This variant contributes to the documented spectrum of PHEX variants, though it remains underrepresented in North African populations. Whole-exome sequencing provided molecular confirmation in our case; however, targeted gene panels or Sanger sequencing of PHEX remain cost-effective first-line options when clinical suspicion is high. The choice of genetic testing strategy should be guided by local resources and pretest probability. Genomic technologies enable definitive diagnostic confirmation, precise familial genetic counseling, and access to targeted therapies [5,6,10].

Conventional treatment with oral phosphate and alfacalcidol improves bone mineralization but does not address the primary pathophysiological mechanism. Burosumab (Crysvita®), a monoclonal anti-FGF23 antibody approved by the FDA and EMA for children over one year of age, represents a major therapeutic advance [1,7,11]. However, its access remains limited in middle-income countries such as Morocco, where institutional reimbursement pathways must be established.

## Conclusions

This case illustrates the typical presentation of XLH confirmed by molecular genetics. Whole-exome sequencing complemented systematic clinical investigation and enabled precise genetic counseling. Early recognition and multidisciplinary management remain essential to optimize outcomes and prevent irreversible orthopedic complications.

## References

[1] Carpenter TO, Whyte MP, Imel EA (2018). Burosumab therapy in children with X-linked hypophosphatemia. N Engl J Med.

[2] Gaucher C, Walrant-Debray O, Nguyen TM, Esterle L, Garabédian M, Jehan F (2009). PHEX analysis in 118 pedigrees reveals new genetic clues in hypophosphatemic rickets. Hum Genet.

[3] Richards S, Aziz N, Bale S (2015). Standards and guidelines for the interpretation of sequence variants: a joint consensus recommendation of the American College of Medical Genetics and Genomics and the Association for Molecular Pathology. Genet Med.

[4] Haffner D, Emma F, Eastwood DM (2019). Clinical practice recommendations for the diagnosis and management of X-linked hypophosphataemia. Nat Rev Nephrol.

[5] Mäkitie O, Doria A, Kooh SW, Cole WG, Daneman A, Sochett E (2003). Early treatment improves growth and biochemical and radiographic outcome in X-linked hypophosphatemic rickets. J Clin Endocrinol Metab.

[6] Beck-Nielsen SS, Brock-Jacobsen B, Gram J, Brixen K, Jensen TK (2009). Incidence and prevalence of nutritional and hereditary rickets in southern Denmark. Eur J Endocrinol.

[7] Gattineni J, Bates C, Twombley K (2009). FGF23 decreases renal NaPi-2a and NaPi-2c expression and induces hypophosphatemia in vivo predominantly via FGF receptor 1. Am J Physiol Renal Physiol.

[8] Quarles LD (2003). FGF23, PHEX, and MEPE regulation of phosphate homeostasis and skeletal mineralization. Am J Physiol Endocrinol Metab.

[9] Rothenbuhler A, Schnabel D, Högler W, Linglart A (2020). Diagnosis, treatment-monitoring and follow-up of children and adolescents with X-linked hypophosphatemia (XLH). Metabolism.

[10] Kinoshita Y, Fukumoto S (2018). X-linked hypophosphatemia and FGF23-related hypophosphatemic diseases: prospect for new treatment. Endocr Rev.

[11] Whyte MP, Zhang F, Wenkert D (2019). X-linked hypophosphatemia: skeletal effects of disease in adults. J Bone Miner Res.

